# Novel Functionalized Boron Nitride Nanosheets Achieved by Radiation-Induced Oxygen Radicals and Their Enhancement for Polymer Nanocomposites

**DOI:** 10.3390/molecules28083444

**Published:** 2023-04-13

**Authors:** Xin Yang, Bingling Zhao, Liudi Ji, Peng Hu, Xiaoming Zhu, Zeyu Li

**Affiliations:** 1 Hubei Key Laboratory of Radiation Chemistry and Functional Materials, Hubei University of Science and Technology, Xianning 437100, Chinazhuxiaoming@hbust.edu.cn (X.Z.); 2 School of Nuclear Technology and Chemistry & Biology, Hubei University of Science and Technology, Xianning 437100, China

**Keywords:** boron nitride nanosheets, surface treatments, interface/interphase, electron beam irradiation

## Abstract

Boron nitride nanosheets (BNNSs) exfoliated from hexagonal boron nitride (h-BN) show great potential in polymer-based composites due to their excellent mechanical properties, highly thermal conductivity, and insulation properties. Moreover, the structural optimization, especially the surface hydroxylation, of BNNSs is of importance to promote their reinforcements and optimize the compatibility of its polymer matrix. In this work, BNNSs were successfully attracted by oxygen radicals decomposed from di-tert-butylperoxide (TBP) induced by electron beam irradiation and then treated with piranha solution. The structural changes of BNNSs in the modification process were deeply studied, and the results demonstrate that the as-prepared covalently functionalized BNNSs possess abundant surface hydroxyl groups as well as reliable structural integrity. Of particular importance is that the yield rate of the hydroxyl groups is impressive, whereas the usage of organic peroxide and reaction time is greatly reduced due to the positive effect of the electron beam irradiation. The comparisons of PVA/BNNSs nanocomposites further indicate that the hydroxyl-functionalized BNNSs effectively promote mechanical properties and breakdown strength due to the enhanced compatibility and strong two-phase interactions between nanofillers and the polymer matrix, which further verify the application prospects of the novel route proposed in this work.

## 1. Introduction

In the past few years, two-dimensional (2D) materials have been intensively exploited due to their unique inherent structures and properties in contrast to those of their bulk and other dimensional counterparts [1,2]. Particularly, h-BN with a layered structure analogous to graphite has been widely studied due to its features such as an ultra-flat surface and a highly stable structure as well as remarkable thermal conductivity and insulating properties [3,4,5]. Moreover, BNNSs exfoliated from h-BN with ultra-thin sheet thickness and ultra-high specific surface area do indeed exhibit great potential in a broad range of applications [6,7,8,9,10,11], such as photonic devices, energy storage and conversion, thermal management, and biomedicines.

The development of advanced polymer-based nanocomposites combined with the unique properties of BNNSs is a non-negligible strategy to realize these applications [12,13,14]. For instance, Tan et al. presented a highly flexible and sensitive biosensor based on thermoplastic polyurethane (TPU) nanocomposites with advanced thermal management capability due to the incorporation of close-contacted BNNSs [15]. Nevertheless, the reinforcement of BNNSs in nanocomposites is always limited by the aggregation of nanofillers, owing to bad compatibility. Surface modifications to BNNSs, including non-covalent coating and covalent functionalization, are alternative ways to address this issue. Non-covalent modifications with surfactants and coating polymers are a feasible route to improve the compatibility of BNNSs in polymer composites without chemical bonding [7,16]. For instance, polymer composites loaded with BNNSs coated with polydopamine (PDA) possess much higher breakdown strength compared with its counterpart with unmodified BNNSs [17,18].

In addition, covalent functionalization is another non-negligible method to optimize the structure and properties of BNNSs. Different from non-covalent modification, the covalent functionalization of BNNSs involves building a direct bond between the functional groups and the h-BN lattices with the sacrifice of B-N bonds. In detail, the covalent functionalization of BNNSs can be divided into two types: one is the edge functionalization of nanosheets, and the other is surface functionalization. The edge functionalization of BNNSs can be achieved under some mild conditions, owing to the inherent structural defects at the edges of nanosheets [19]. For example, Lin et al. reported that simple sonication in water can graft -OH groups onto the edges of BNNSs [20], and the resulting hydroxylated BNNSs show stable water solubility. Ball milling can also facilitate edge grafting of BNNSs with functional groups (-OH, -NH_2_ etc.), depending on the chemical reagents used in the processes [4,6,21]. In comparison, due to the high chemical stability of the hexagonal lattice in h-BN, the surface hydroxylation of BNNSs is generally realized with strongly oxidizing agents under high temperature and pressure [22]. For example, the surface hydroxylation of BNNSs can be achieved after a hydrothermal reaction with H_2_NO_3_ at 200 °C for 24 h [17]. Another important method discovered by Sainsbury et al. is the radical oxidation of BNNSs using organic peroxides (di-*tert*-butylperoxide (TBP)) [23] or organo-azide (4-methoxybenzyloxycarbonyl azide) [24] under high temperatures. In fact, the route that the radical oxidation of BNNSs follows, the treatment using piranha solution, is still the mainstream way to prepare surface-hydroxylated BNNSs [25]. Due to the higher grafting rate compared with their counterparts with edge functionalization, the surface covalent modification of BNNSs with hydrophilic groups was identified as able to improve solution processability as well as miscibility and compatibility with polymers, and it is used as an intermediate for further preparation of BN-based derivative materials to meet the needs of different application fields [5].

In this work, we paved a green and scalable route to surface hydroxylate BNNSs with high energy electron beam irradiation. As shown in Figure S1, exfoliated BNNSs were first attracted to *tert*-butoxy radicals decomposed from TBP induced by irradiation to yield alkoxylated BNNSs (denoted as BNNSs-TB@x, where x is the irradiation dose). The second step was the hydrolysis of the bonded alkoxy groups with piranha solution to prepare hydroxylated BNNSs (denoted as BNNSs-OH@x). The outcomes show that as-prepared BNNSs-OH possesses abundant surface hydroxyl groups as well as good structural integrity. It is worth pointing out that the yield rate of surface hydroxyl groups in this work is encouraging compared to previous reports, and the used amount of organic peroxide and the reaction time was greatly reduced. Further in-depth study found that BNNSs-OH showed excellent water solubility and significantly improved the mechanical properties and breakdown strength of polyvinyl alcohol (PVA), which confirms the commercial prospects of the hydroxyl-functionalized BNNSs prepared under electron beam irradiation.

## 2. Results and Discussion

**Figure 1:**
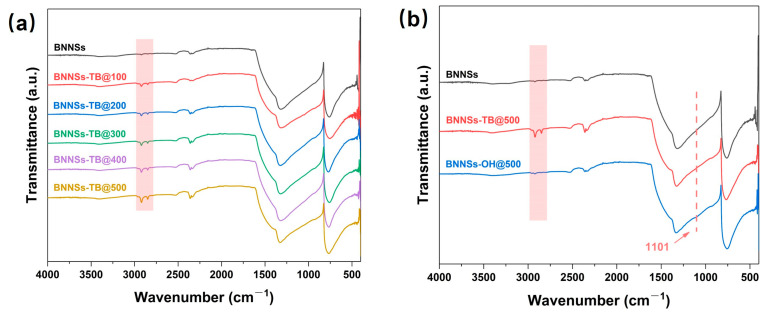
(**a**) FT-IR spectra of BNNSs-TB with different doses. (**b**) FT-IR spectra of BNNSs, BNNSs-TB@500, and BNNSs-OH@500.

XRD patterns of BNNSs and bulk h-BN are shown in Figure S2, where it can be seen that the pattern of BNNSs and h-BN present the same characteristic peaks. The enlarged pattern from 10° to 40° indicates that the main peak of BNNSs shows a broadened width with a shorter height, indicating a decreased number of layers in the nanosheets [26]. FT-IR was used to characterize the functionalization procedures of the BNNSs. As shown in Figure 1a, compared with unmodified BNNSs, the appearance of absorption peaks at 2852 and 2923 cm^−1^ in BNNSs-TB are ascribed to the symmetric and asymmetric stretching of the methyl groups [27]. In addition, these peaks became obvious with continuous doses; this is reasonable because more oxygen radicals can easily be decomposed from TBP under higher doses, thereby leading to increased grafting groups. Not only that, the B-N vibration found at 760 and 1321 cm^−1^ in the spectrum of pristine BNNSs was shifted upfield to 769 and 1331 cm^−1^ following *tert*-butoxy functionalization, which was due to the lattice vibrations that resulted from grafting the bulky tertbutyl substituents [23]. These results demonstrate that organic radicals successfully attacked BNNSs under electron beam irradiation, forming tert-butoxy groups. The comparation of the FT-IR spectra of BNNSs, BNNSs-TB@500, and BNNSs-OH@500 are given in Figure 1b. It is clear that the stretching peaks of the methyl groups were almost absent in the spectrum of BNNSs-OH@500, whereas a new slight peak appeared at 1101 cm^−1^, which represents the in-plane bending of B-OH, confirming the presence of the B-OH functional groups [23]. In addition, there was a faint and flat peak at 3400 cm^−1^, which was assigned to the hydroxyl group. In fact, as shown in Figure S3, the absorption peaks of hydroxyl groups almost all appeared in the spectra of BNNSs-OH prepared under different doses, but the intensities of the peaks are not convincing. Therefore, it is necessary to further confirm the presence of hydroxyl groups in the surface of the BNNSs-OH and calculate the yield rate of the functional groups.

**Figure 2:**
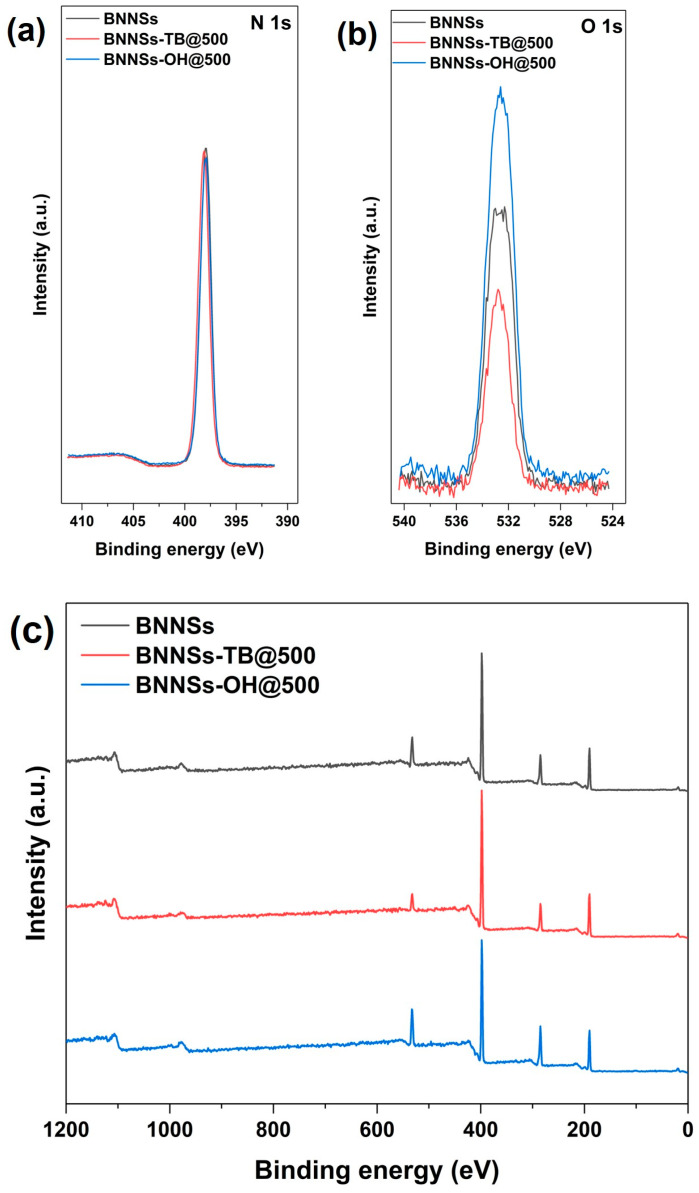
(**a**) N 1s peak in XPS spectra, (**b**) O 1s peak in XPS spectra, and (**c**) XPS spectra of BNNSs, BNNSs-TB@500, and BNNSs-OH@500.

**Figure 3:**
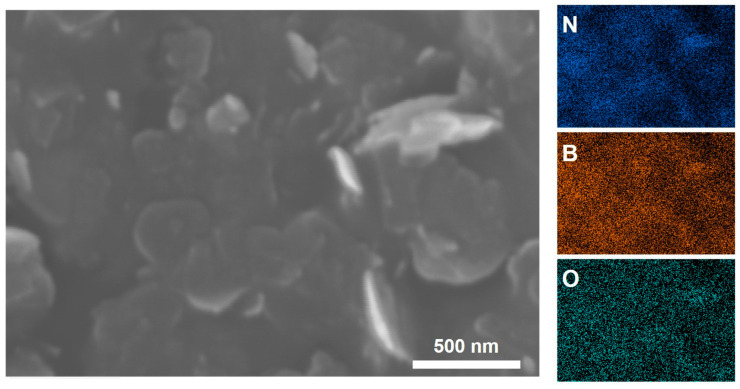
EDS mapping of N, B and O elements in BNNSs-OH@500.

XPS was performed to characterize the changes of element content in BNNSs, BNNSs-TB@500, and BNNSs-OH@500. As shown in Figure 2, the XPS spectra of three different samples all identified the B 1s and N 1s peaks at 190.9 and 397.9 eV [22,28], respectively, and they showed the same peak height at N 1s (Figure 2b). As expected, the O 1s spectrum of BNNSs-OH@500 in Figure 2a possessed the highest peak height at 532.6 eV, whereas the corresponding spectrum of pristine BNNSs showed a relatively weak peak at 532.3 eV. This residual oxygen signal is speculated to stem from the trace DMF which was trapped within interstitial voids in exfoliated nanosheets [23]. The enhanced oxygen signal in the spectrum of BNNSs-OH@500 encourages the notion that hydroxyl functionalization of BNNSs occurred. In Figure 3, EDS-layered images of BNNSs-OH@500 also provide similar results. It is clear that an abundant oxygen signal spread over the entire scan area, and the concentration of the oxygen signal was highly coincident with the N and B signals. This feature proves the abundant oxygen elements on the surface of BNNSs-OH@500. According to the FT-IR analysis results, XPS, and EDS, it can be inferred that the hydroxyl group was successfully grafted to the surface of BNNSs after functionalization procedures.

**Figure 4:**
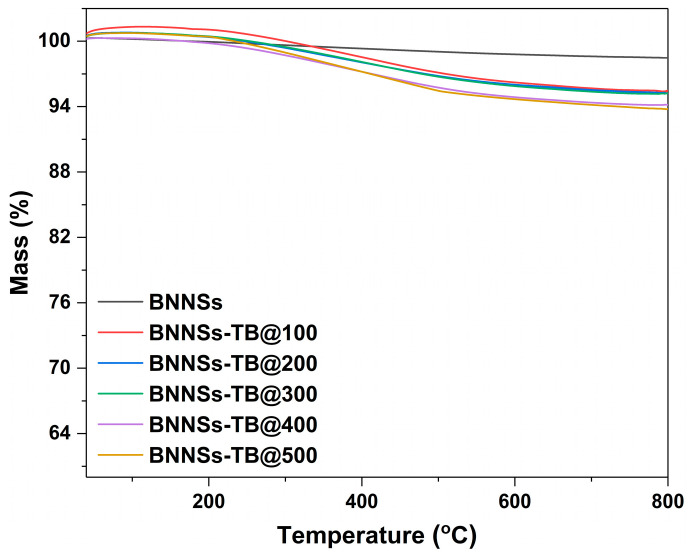
TG curves of BNNSs-TB with different doses.

TG analysis of the purified and dried BNNSs-TB prepared with different doses was conducted to quantify the organic fraction grafted to the BNNSs. As shown in Figure 4, the mass loss of BNNSs-TB grew with irradiation doses. In addition, the plot of BNNSs-TB@500 shows the highest mass loss was 6.2%, whereas only a very slight mass loss can be observed in the curve of unmodified BNNSs until reaching a temperature of ∼800 °C. It is speculated that the significant decrease in mass loss between 200 and 400 °C can be attributed to the pyrolysis of covalently grafted organic tert-butoxy groups. Taking the mass loss of 6.2% into account, the BNNSs-TB@500 yielded a functionalization of ∼2.7% of boron atoms in the h-BN lattice. This grafting rate is comparable to that (about 4%) obtained by the representative hydrothermal method [23], whereas the amount of TBP used in this work is only one-tenth of its counterpart. We speculate that the improved yield rate and reduced usage of organic chemicals in this work was due to the affluent oxygen radicals decomposed from TBP under irradiation. Meanwhile, the high energy of the electron beam also helped promote the breaking of B-N bonds and further increase the grafting ratio. Considering that the time-consuming organic radical oxidation procedure for BNNSs-TB@500 took only about 2.5 h, whereas the previous reported hydrothermal reaction needed 12 h at 120 °C, the novel modification method of BNNSs incorporated with electron beam irradiation, a feasible and mature commercial technology [29,30], will obviously have better economic prospects.

**Figure 5:**
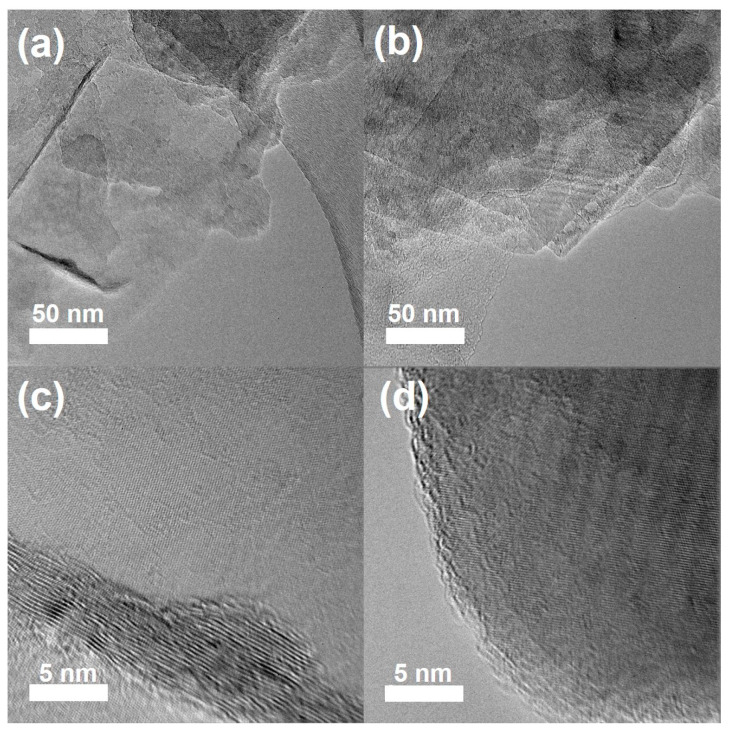
TEM images of (**a**) BNNSs and (**b**) BNNSs-OH@500. HRTEM images of (**c**) BNNSs and (**d**) BNNSs-OH@500.

SEM and TEM were used to assess the structural integrity of the functionalized BNNSs after the radiation-induced oxygen radical reaction. As shown in Figure S3, the SEM images prove that the modified BNNSs still maintained a two-dimensional structure, and the lateral dimensions of BNNSs-TB@500 and BNNSs-OH@500 were of about 1 μm, which is almost unchanged from that of the pristine exfoliated BNNSs. More importantly, this demonstrates that the layered structure of modified BNNSs did not suffer obvious damage, such as holes or fragmentation, during the functionalization process, even under the irradiation dose of 500 kGy. The representative TEM images of pristine BNNSs and of BNNSs-OH@500 are given in Figure 5 to further confirm the lattice’s integrity. It can be seen that the pristine BNNSs had an ultrathin nanosheet structure with curled edges, and the hydroxyl-modified BNNSs possessed similar mono- and few-layered structures. The HRTEM images show that the lattice of BNNSs-OH@500 remained intact without obvious defects compared with that of exfoliated BNNSs. Because the 2D structure is an indispensable prerequisite for the unique performance of BNNSs [3], the structural integrity of the functionalized BNNSs in this work is helpful to facilitate its application in related fields.

**Figure 6:**
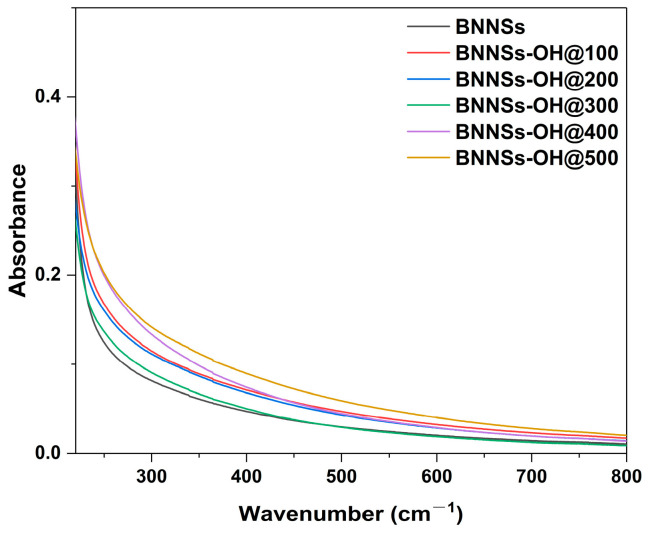
UV-vis spectra of the equilibrated aqueous solution of BNNSs and BNNSs-OH with different doses.

It is widely accepted that the hydroxylation of inorganic nanoparticles will significantly optimize their water solubility. Therefore, UV-vis spectroscopy was carried out to investigate the change in water solubility of the BNNSs-OH relative to unmodified BNNSs. As before, BNNSs-OH and pristine BNNS samples were dispersed in deionized water with a concentration of 1 mg/mL via bath sonication for 12 h. To remove any aggregated and insoluble composition, the supernatant was then centrifugated at 10,000 rpm for 30 min and allowed to equilibrate by standing for 24 h before analysis. The UV-vis spectra of the as-equilibrated BNNSs-OH and BNNS solution are shown in Figure 6. It was found that the spectra of BNNSs-OH had substantially increased absorbencies compared to pristine BNNSs, and the spectrum of BNNSs-OH@500 showed the highest absorbance intensity among all samples. This indicates that BNNSs-OH@500 possesses higher water solubility in comparison to other hydroxylated BNNSs and pristine BNNSs. This difference in water solubility suggests that there are more grafted hydroxyl groups on BNNS-OH@500. The enhanced water solubility is also demonstrated by the optical photograph of BNNSs and BNNSs-OH aqueous solution shown in Figure S5. It is clear that the BNNSs-OH@500 aqueous solution shows a darker color, indicating its higher soluble concentration. In addition, the obvious Tyndall effect visible in Figure S5b also certifies the uniform dispersion of modified BNNSs.

Recently, polymer-based composites with BNNSs have attracted increasing attention on account of its attractive mechanics, thermal conductivity, and electrical insulation. In this work, three different PVA-based nanocomposites, including PVA/BNNSs, PVA/BNNSs-OH, and cross-linked PVA/BNNSs-OH (denoted as cPVA/BNNSs-OH), were prepared to make good use of the hydroxylated BNNSs and analyze the effect of hydroxylation on its reinforcement. PVA was incorporated as a host due to its abundant hydroxyl groups and good water solubility. Due to the barren surface of the unmodified BNNSs, there was no obvious interfacial interaction between pristine BNNSs and PVA matrices in PVA/BNNSs nanocomposites. However, as illustrated in Figure S4, the two-phase interactions in PVA/BNNSs-OH and cPVA/BNNSs-OH nanocomposites cannot be ignored. The former mainly consists of hydrogen bonding between the hydroxyl groups on the molecular chain of PVA and the BNNSs-OH surface, whereas cPVA/BNNSs-OH comprises cross-linked networks consisting of PVA and BNNSs-OH bonded with GA [31]. The cross-linked structure can be confirmed via FT-IR spectra, as shown in Figure S7. The large bands at approximately 3262 cm^−1^ can be attributed to –OH groups associated with the stretching vibration in PVA and BNNSs-OH. Compared with the PVA/BNNSs-OH nanocomposites, the spectrum of cPVP/BNNNSs-OH displays a decreased intensity of –OH, which means that more of the –OH groups were involved in the cross-linking reaction with C=O from GA. In addition, the bands at 1380 cm^−1^ ascribed to C-O-C also indicate the cross-linked structure.

**Figure 7:**
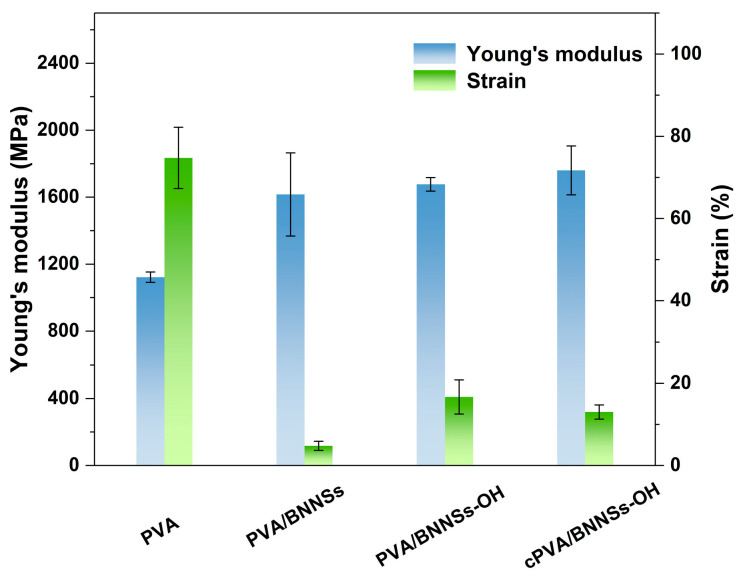
Young’s modulus and strain of PVA, PVA/BNNSs, PVA/BNNSs-OH, and cPVA/BNNSs-OH films.

With the incorporation of different BNNSs nanofillers, the mechanical, thermal conductivity, and insulation properties of PVA nanocomposites were investigated. Figure 7 shows the evaluated Young’s modulus (*Y*) and strain-to-break (*ε*_b_) of various nanocomposites. It can be seen that the Young’s modulus of the nanocomposites increased with the addition of nanofillers. It is not surprising that the addition of BNNSs, a stiff material, increased the *Y* of the PVA matrix. In addition, cPVA/BNNSs-OH possessed the highest *Y* of 1760 MPa, which is 57% higher than that of pristine PVA. This is because the cross-linked networks in cPVA/BNNSs nanocomposites have the strongest chemical bonds of these three different nanocomposites. Another important feature is that PVA/BNNSs are more brittle, and its *ε*_b_ was only 4.77%, which is much lower than that of pristine PVA (about 74%). This is attributed to the aggregation of BNNSs in polymer composites due to their poor compatibility. Nevertheless, based on the enhanced compatibility of BNNSs-OH, the *ε*_b_ of PVA/BNNSs-OH and cPVA/BNNSs-OH increased to 16.7% and 13.0%, respectively. It was reasoned that the *ε*_b_ of PVA/BNNSs-OH and cPVA/BNNSs-OH are still lower than that of pristine polymer matrix. The strong intermolecular interactions existing in these two composites limit the mobility of PVA molecular chains, thereby leading to this decreased *ε*_b_.

Because the biggest advantage of BNNSs is their high thermal conductivity, it is informative to examine the differences of thermal conductivity among PVA-based nanocomposites. As shown in Figure S8, the pristine PVA showed a bad thermal conductivity level of 0.178 W m^−1^ K^−1^. However, the loading of BNNSs significantly enhanced thermal conductivity. and the cPVP/BNNSs-OH nanocomposite showed the highest value of 0.216 W m^−1^ K^−1^, which is 20% higher than that of pristine PVP. We speculate that the superior thermal conductivity in the cross-linked nanocomposite was due to enhanced compatibility and interfacial interaction. Considering that the content of BNNSs in the nanocomposites was only 2 wt%, the increment in thermal conductivity is still impressive.

**Figure 8:**
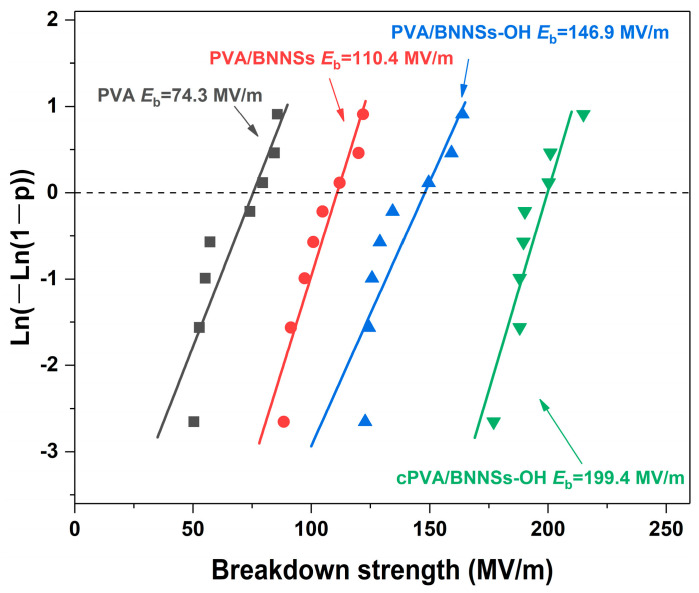
Weibull breakdown strength distributions of PVA, PVA/BNNSs, PVA/BNNSs-OH, and cPVA/BNNSs-OH films.

The breakdown strength (*E*_b_) of raw PVA and of PVA-based nanocomposites was analyzed using the two-parameter Weibull statistical distribution, which is given as P(*E*) = 1 − exp((−*E*/*E*_b_)*β*). In this function, P(*E*) is the cumulative probability of electrical failure, *E* is the measured value of breakdown strength, *E*_b_ represents the characteristic Weibull breakdown strength (which means the sample has a probability of 63.2% to breakdown under the applied electrical field), and *β* is used to evaluate the dispersion of data [32,33]. In this work, at least eight samples were tested for each Weibull fitting. The statistical *E*_b_ values of different samples are given in Figure 8. It is clear that the nanocomposites show increased *E*_b_ values with the loading of BNNSs nanofillers. For instance, the *E*_b_ of pristine PVA was only 74.3 MV/m, whereas those of PVA/BNNSs and PVA/BNNSs-OH were 110.4 MV/m and 149.6 MV/m, respectively. As expected, the cPVA/BNNSs-OH nanocomposite possessed the highest *E*_b_ of 199.4 MV/m, which is 168% higher than that of pristine PVA.

**Figure 9:**
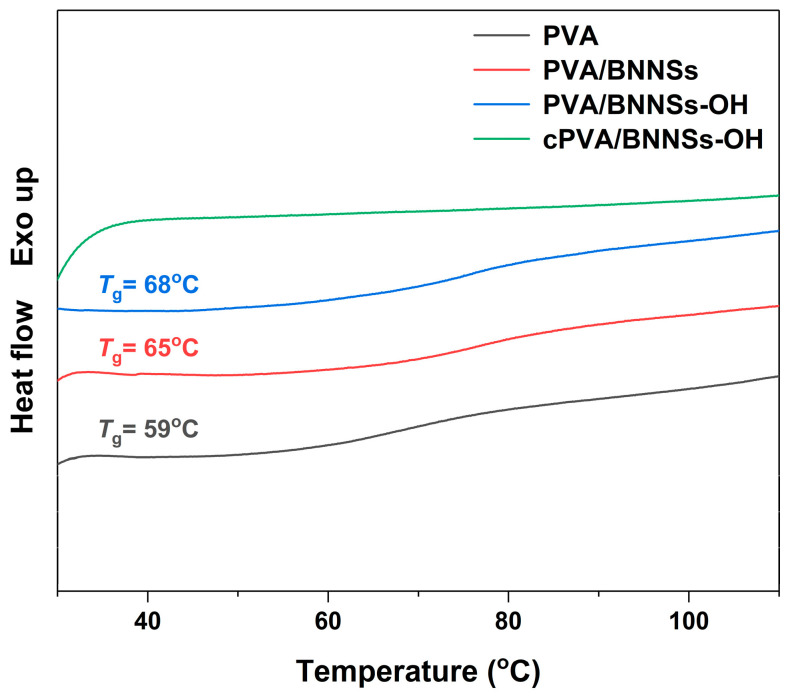
DSC analyses of PVA, PVA/BNNSs, PVA/BNNSs-OH, and cPVA/BNNSs-OH.

**Figure 10:**
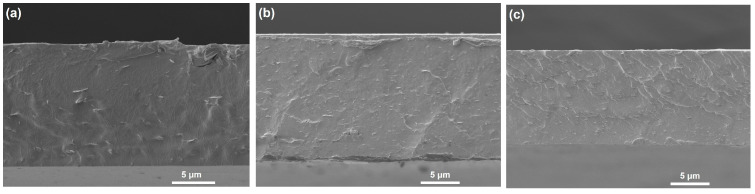
Cross-section SEM images of (**a**) PVA/BNNSs, (**b**) PVA/BNNSs-OH, and (**c**) cPVA/BNNSs-OH nanocomposites.

The largely enhanced *E*_b_ in cross-linked nanocomposites was attributed to the following two aspects. First, due to the inherent high insulation of BNNSs, the injected carriers will greatly reduce even under high electric field [34,35]. More importantly, the incorporation of modified BNNSs effectively limits the movement of molecular chain segments in PVA, thereby leading to an enhanced *E*_b_ [36]. The DSC analysis in Figure 9 successfully confirms this hypothesis. The *T*_g_ of PVA was 59 °C, whereas those of PVA/BNNSs and PVA/BNNSs-OH were 65 °C and 68 °C, respectively. Interestingly, there was no obvious glass transition behavior in the DSC curve of the cPVA/BNNSs-OH nanocomposite. We speculate that this is because the cross-linking networks between PVA and BNNSs-OH greatly restrict the movement of the PVA molecular chain. Second, as the cross-section SEM images in Figure 10 indicate, the pristine BNNSs aggregates in the PVA/BNNSs nanocomposites, and there are visible pores in the interfaces between the nanofillers and the matrix. However, the hydroxylated BNNSs homogeneously dispersed in PVA/BNNSs-OH and cPVA/BNNSs-OH nanocomposites without macroscopic structural defects. This enhanced interface interaction thereby improves the mechanical and insulating properties of nanocomposites [37].

## 3. Materials and Methods

### 3.1. Reagents and Apparatus

h-BN powder (1 μm, 98%) was purchased from Sigma-Aldrich. *N*,*N*-dimethylformamide (DMF), di-*tert*-butylperoxide. and acetonitrile of analytical grade were obtained from Aladdin Reagent Limited Corporation. Sulfuric acid (98%), hydrochloric acid (36~38%), hydrogen peroxide solution (28%), and toluene were obtained from Sinopharm Chemical Reagent Co., Ltd. (Shanghai China). Polyvinyl alcohol (PVA) and glutaraldehyde (GA, 50% aqueous solution) were supplied by Tansoole (Shanghai, China).

### 3.2. Exfoliation of BNNSs

Exfoliated BNNSs were prepared according to previous works [38,39]. Briefly, 6 g h-BN powders were dispersed in 500 mL DMF with vigorous stirring for 2 h followed by bath ultrasonication for 30 h. The resultant mixture was centrifuged at 3000 rpm for 30 min twice to remove unexfoliated powders. The translucent milky supernatant with exfoliated BNNSs was then vacuum filtered with a filter membrane with a pore size of 0.45 μm. After vacuum drying at 60 °C for 24 h, exfoliated BNNSs were obtained.

### 3.3. Synthesis of Hydroxyl-Functionalized BNNSs

A volume of 60 mL TBP and 0.1 g pristine BNNSs were first mixed in a beaker with vigorous stirring for 6 h, followed by bath ultrasonication for 2 h to ensure sufficient dispersion of the nanosheets. The mixture was then sealed in transparent PE bags (20 cm × 20 cm) and treated with electron beam irradiation with a 1.0 MeV electron accelerator at room temperature. The total irradiation doses of the samples in this work were 100, 200, 300, 400, and 500 kGy with a dose per pass of 20 kGy, and the irradiation time of every pass was 6 min. The obtained suspension was then vacuum-filtered and washed with mass acetonitrile and toluene. The solids were then dried in an oven at 60 °C to get BNNSs-TB. To defunctionalize the BNNSs-TB and yield hydroxyl-functionalized BNNSs, the dried BNNSs-TB was first added to sulfuric acid (98%, 45 mL), stirred for 30 min, and then ultrasonicated for 30 min. Hydrogen peroxide (28%, 15 mL) was then slowly added to the readily dispersed mixture to make up the piranha solution (H_2_SO_4_:H_2_O_2_, 3:1), stirring for 2 h. The suspension containing as-prepared BNNSs-OH was then vacuum-filtered on a membrane filter with a pore size of 0.45 μm and washed with water (2 L) to ensure that any water-soluble residue was removed. The purified BNNSs-OH on the filter was dried using a vacuum oven at 60 °C for 24 h.

### 3.4. Preparation of Polymer Nanocomposites

Three different PVA-based nanocomposites were prepared via the following steps. Briefly, PVA pellets were first dissolved in deionized water with a concentration of 0.05 g/mL at 90 °C for 8 h. Meantime, pristine BNNSs or BNNSs-OH@500 aqueous solutions were prepared in a concentration of 1 mg/mL with stirring for 30 min followed by ultrasonication. Then, the aforementioned PVA- and BNNSs-based aqueous solutions were mixed in a volume ratio of 1:1 with vigorous stirring. The mixture was finally cast onto quartz glass coated with PTFE and dried at 50 °C for 12 h in an oven to completely remove water. The final nanocomposite films were then peeled off of the quartz plates. To obtain cross-linked cPVA/BNNSs-OH nanocomposites, crosslinking agent GA and catalyst HCl solution were added to the PVA/BNNSs-OH mixed aqueous solution before the solution-casting procedure [31]. The molar ratio of GA to the total mole number of hydroxyl groups on the PVA chains was 1:20, and the molar ratio of HCl to GA was 1:5. To conduct the tensile test, sample films were cut into strips of 5 mm in width and 50 mm in length with a thickness of 50 μm, and the thickness of films for breakdown strength test was finely controlled at 15 μm.

## 4. Conclusions

In summary, an easy and new route to covalently hydroxylate BNNSs with improved yield rate and reduced usage of organic reagents was successfully developed. The high energy of electron beam irradiation effectively induced oxygen radicals from TBP, thereby significantly enhancing the surface modification of BNNSs. The as-prepared BNNSs-OH with abundant hydroxyl possessed enhanced water solubility, impressive structural integrity, and optimized compatibility, which further improved its enforcements of the mechanics, thermal conductivity, and insulating properties of the PVA matrix. This work opens a feasible route to facilitate the surface covalent functionalization of BNNSs via electron beam irradiation. Moreover, the boosted performance of polymer nanocomposites with the incorporation of BNNSs-OH will realize a broad range of applications.

## Data Availability

Data will be made available upon reasonable request.

## Supplementary Materials

The following supporting information can be downloaded at: https://www.mdpi.com/article/10.3390/molecules28083444/s1, Figure S1: The schematic of the functionalization procedures of BNNSs under electron beam irradiation (EB); Figure S2: XRD patterns of h-BN and exfoliated BNNSs; Figure S3: FT-IR spectra of BNNSs-OH with different doses; Figure S4: SEM images of (a) BNNSs, (b) BNNSs-TB@500 and (c) BNNSs-OH@500; Figure S5: (a) Optical photograph of BNNSs and different BNNSs-OH aqueous solution, (b) the Tyndall effect in BNNSs-OH@500 aqueous solution; Figure S6: The schematic of two-phase interaction in (a) PVA/BNNSs-OH and (b) cPVA/BNNSs-OH nanocomposites; Figure S7. The FT-IR spectra of PVA and PVA based nanocomposites; Figure S8. The thermal conductivity of PVA and PVA based nanocomposites.
