# Supplementary material for: Novel Functionalized Boron Nitride Nanosheets Achieved by Radiation-Induced Oxygen Radicals and Their Enhancement for Polymer Nanocomposites
Source: Molecules. 2023 Apr 13;28(8):3444. doi: 10.3390/molecules28083444 (PMC10141363; doi:10.3390/molecules28083444)
Supplement: Supplementary file 1 [file molecules-28-03444-s001.zip › molecules-2288786-supplementary.pdf]

Novel Functionalized Boron Nitride Nanosheets Achieved by Radiation-induced Oxygen Radicals and Their Enhancement for Polymer Nanocomposites

Xin Yang [1,2], Bingling Zhao[2], Liudi Ji[1,2,*], Peng Hu[1,2], Xiaoming Zhu[1,2], Zeyu Li [1,2,*]

[1] Hubei Key Laboratory of Radiation Chemistry and Functional Materials, Hubei University of Science and Technology, Xianning 437100, China
[2] School of Nuclear Technology and Chemistry & Biology, Hubei University of Science and Technology, Xianning 437100, China
* Correspondence: lizeyu725@163.com (Z. Li); jiliudi@126.com (L. Ji)

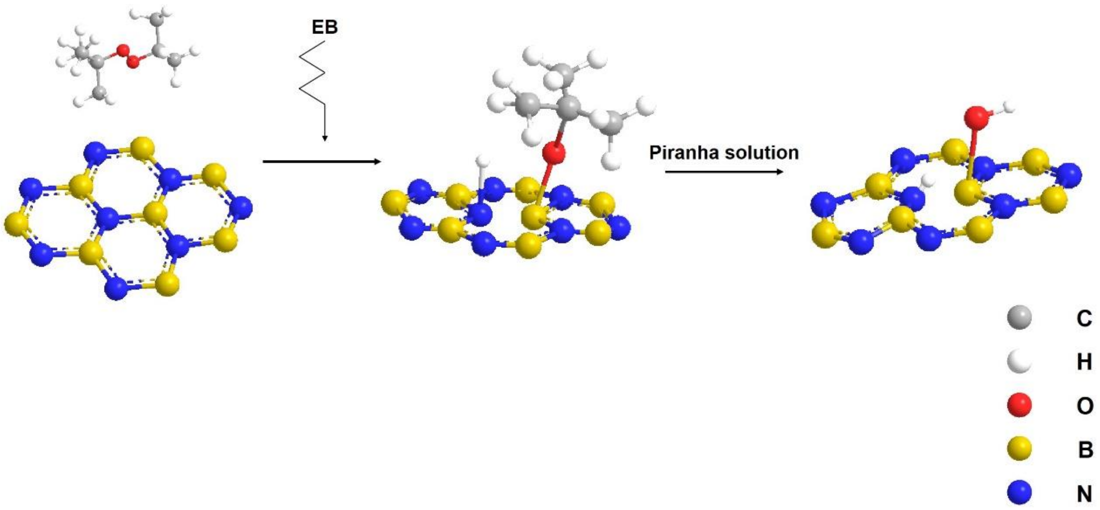

Figure S1. The schematic of the functionalization procedures of BNNSs under electron beam irradiation (EB).

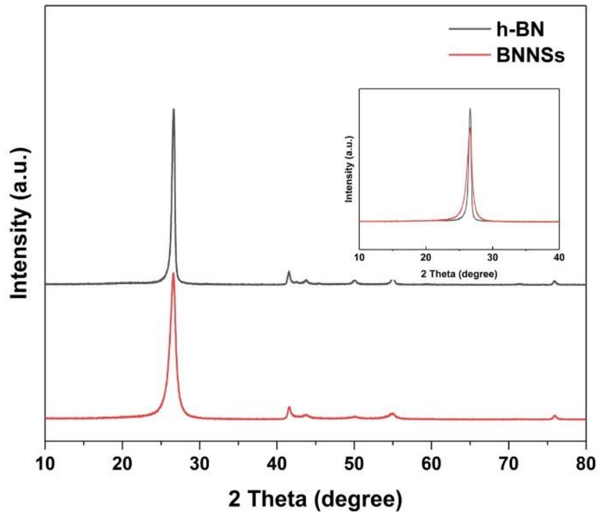

Figure S2. XRD patterns of h-BN and exfoliated BNNSs.

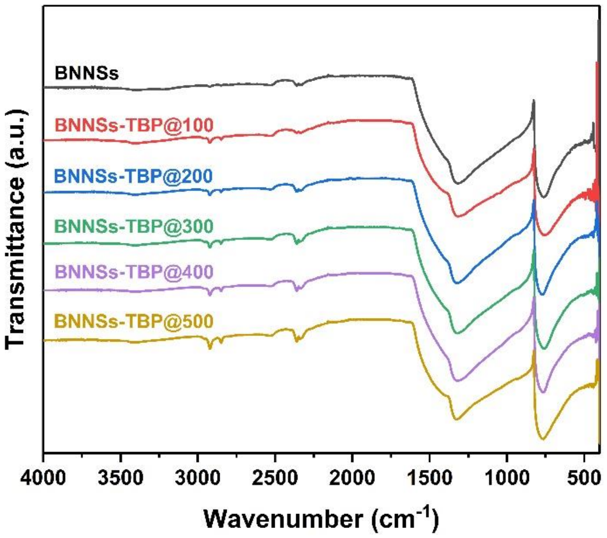

Figure S3. FT-IR spectra of BNNSs-OH with different doses.

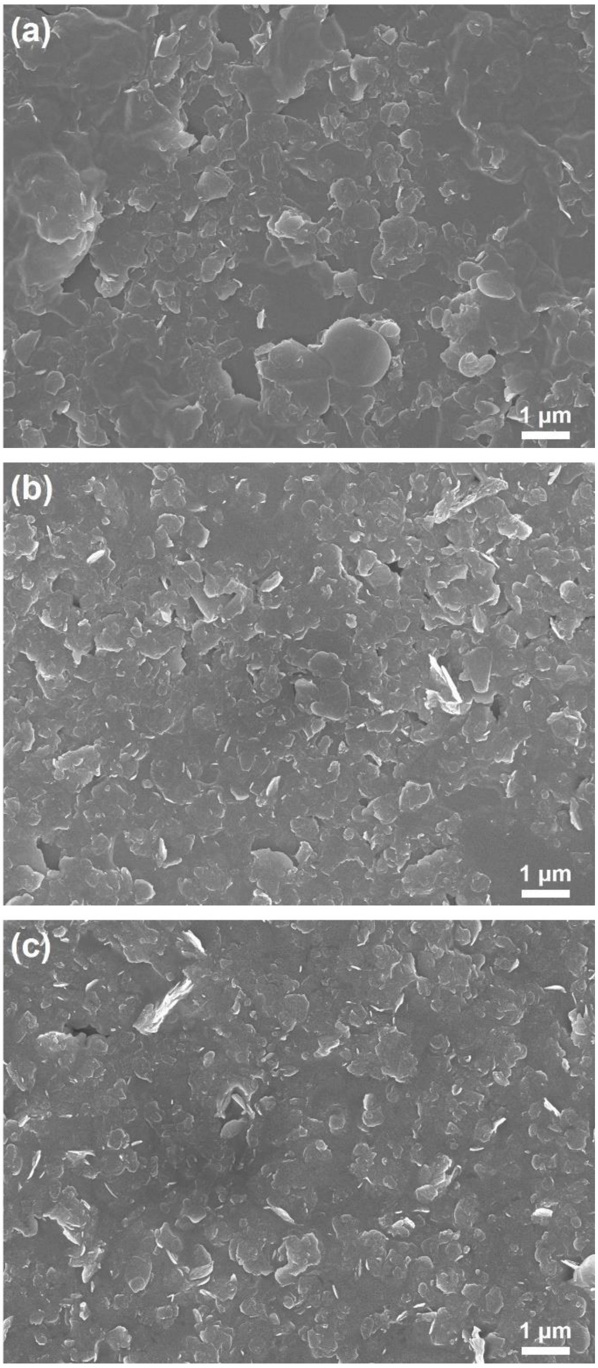

Figure S4. SEM images of (a) BNNSs, (b) BNNSs-TB@500 and (c) BNNSs-OH@500.

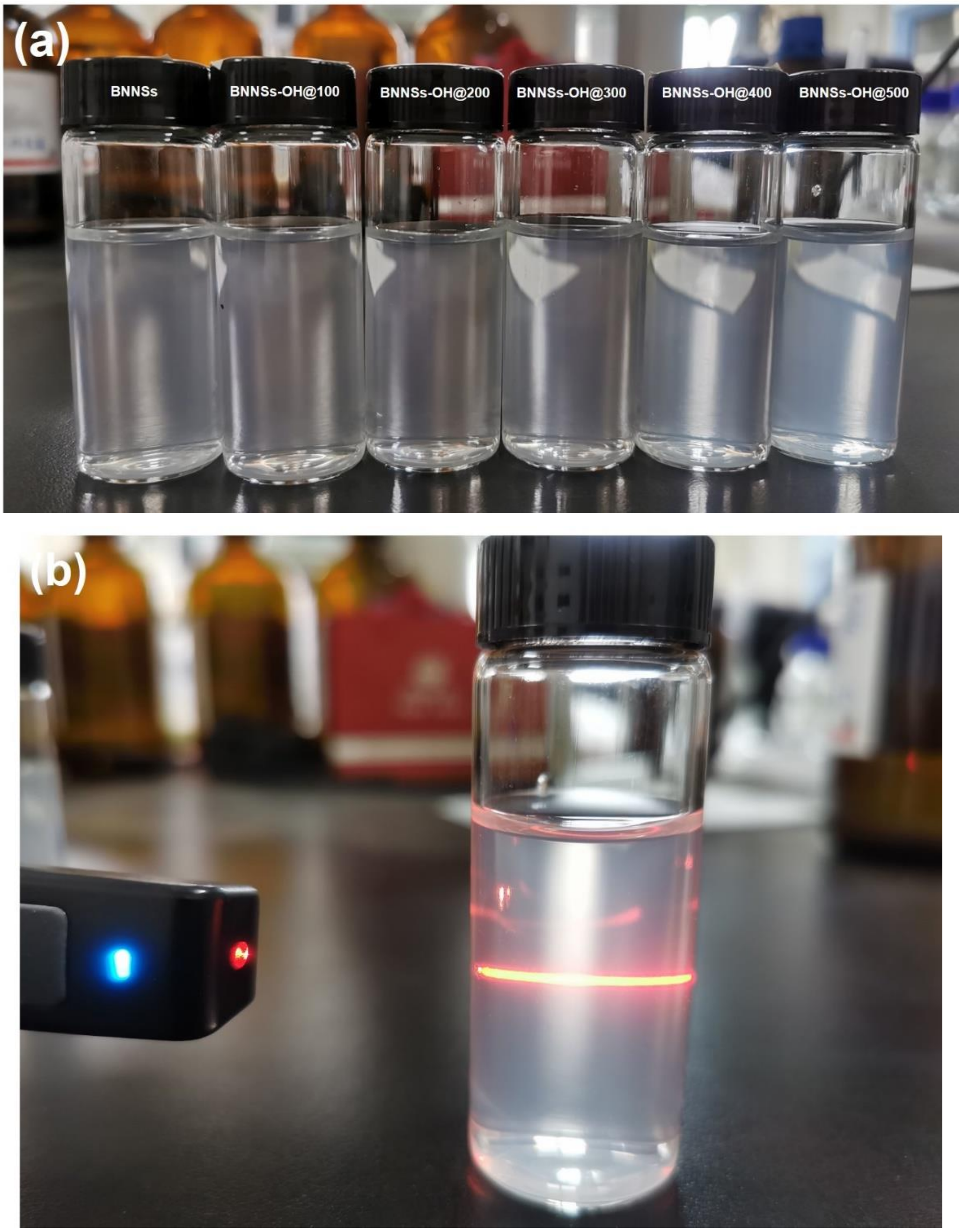

Figure S5. (a) Optical photograph of BNNSs and different BNNSs-OH aqueous solution, (b) the Tyndall effect in BNNSs-OH@500 aqueous solution.

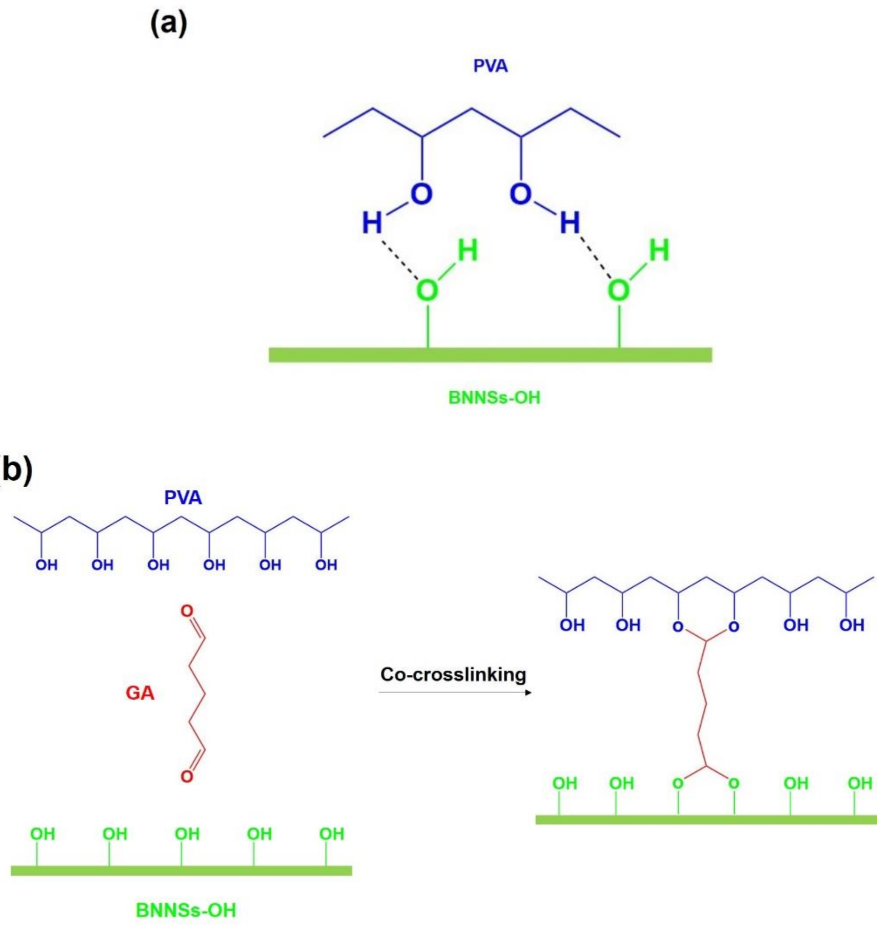

Figure S6. The schematic of two-phase interaction in (a) PVA/BNNSs-OH and (b) cPVA/BNNSs-OH nanocomposites.

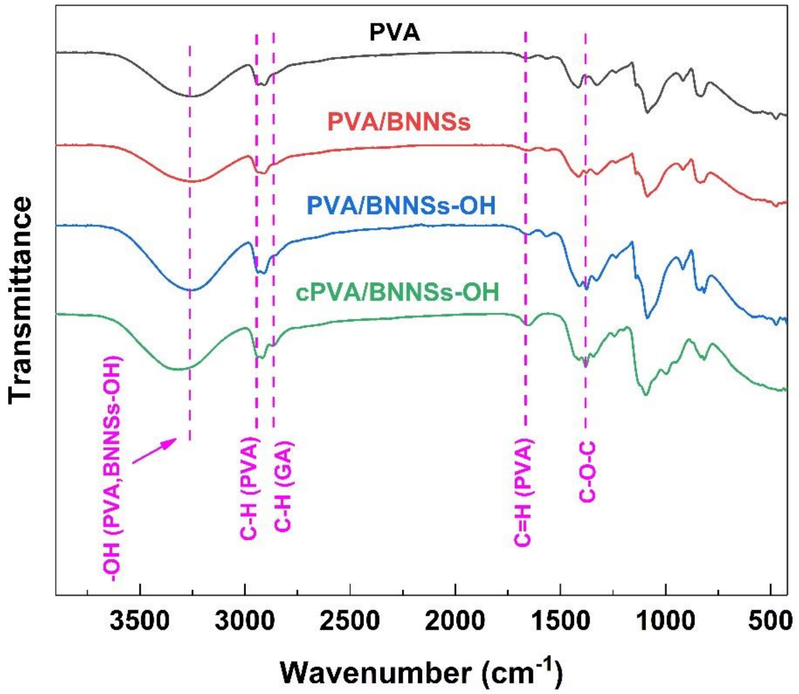

Figure S7. The FT-IR spectra of PVA and PVA based nanocomposites.

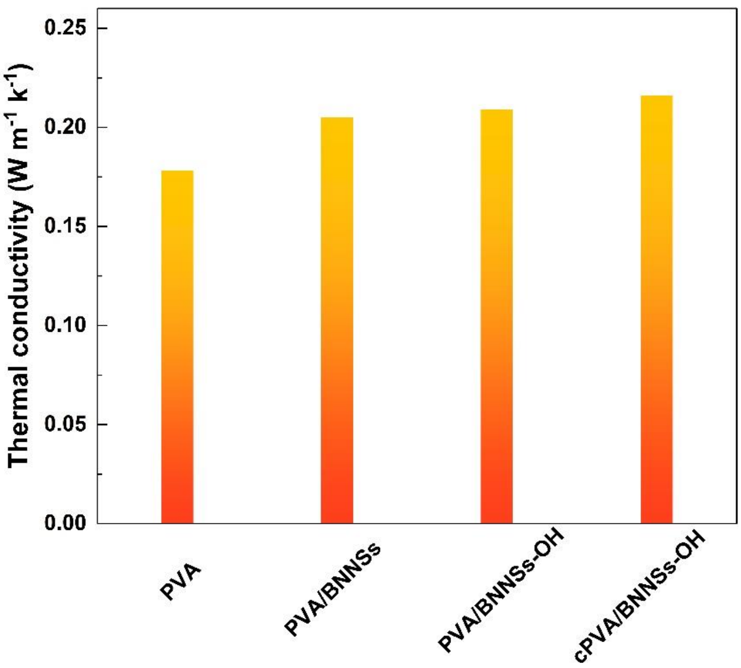

Figure S8. The thermal conductivity of PVA and PVA based nanocomposites.
